# Prosthetic Dentistry

**Published:** 1886-08

**Authors:** James B. Hodgkin


ARTICLE II.

### PROSTHETIC DENTISTRY.

BY JAMES B. HODGKIN, D. D. S.

What is the condition of prosthetic dentistry in this country at the present time?

Although this question has been often answered in papers and reports of investigations read before dental societies, in discussions of the same, and in articles published in our journals, it is proposed again to call attention to it, and to continue to do so until the profession is induced, if possible, to remedy existing faults in that direction, which, in some cases, come very near being, if they are not, crimes.

The broad statement is made in the beginning, that while operative dentistry has, in the treatment of decayed teeth and diseases of the oral cavity, made giant strides and almost incredible progress, so far as both science and art are concerned, in the last thirty or forty years, prosthetic dentistry has, in both these respects, rather retrograded, than advanced, with the mass of practicing dentists. It cannot be truthfully denied that praise and credit for the former, as well as blame for the latter, belong to the dental profession ; and it is a duty incumbent now, as it always has been, on its members, to remove the just cause for this blame.

The true cause for this seems to lie, and doubtless does lie, in the fact that in prosthetic dentistry the great study, as a general rule, has been to produce the cheapest possible substitutes for the natural teeth and oral tissues lost by the ravages of decay and disease. In this effort both art and science have been sacrificed to cheapness and ease of manipulation. In the face of the rule that "the beauty of art is to conceal art," there are comparatively very few cases in which artificial teeth in the mouth cannot be detected as such at a glance by the most casual observer. The teeth themselves are caricatures of the natural organs, not adapted in color, shape or arrangement, to the physiognomy of the wearer, while in the formation of the plates to which they are attached, no attention is paid to the restoration of the original and natural form and appearance of the face.

But this offence against art and esthetics is not so grave as that which, in the pursuit of cheapness, or in supplying the demand on the part of the mass of the people for cheap dental plates, is regardless and reckless of the pathological effects of some of the cheap materials used for that purpose.

It is very interesting to any one who has the inclination (I was going to say, and time ; but if the inclination exists, the time can be found) to investigate this subject, having before him its conditions forty years ago, with the substances since proposed for the material used for " bases " (to use a modern term) for artificial teeth at that time, and the consequent different manipulation required for each substitute. Gold was then *the* material for dental plates, platinum being used very rarely, while in many cases temporary plates were made of silver. The latter metal, however, was objectionable even for temporary plates, on account of its ready conversion into salts of silver in the mouth.

The two former have to this day held their places as being entirely unobjectionable, in a hygenic point of view, for any form of dental appliance, even though the gold should be alloyed with silver and copper down to an 18-carat quality. It is universally admitted that these two metals and porcelain do not produce any deleterious effects on the oral structures and tissues, nor on the system generally. With the exception of pure block tin and a very few alloys formed into plates by casting, all the substitutes offered for these are more or less injurious, either locally or generally, or both.

While some alloys composed of base metals, as, for instance, Blandy's cheoplasty, have gone entirely out of use, on account of the chemical changes they undergo in the mouth, no metal or alloy formed into plates by casting, however unobjectionable in that respect, has come into general use, because of the uncertainty of making a good casting and the skill and experience required to overcome that difficulty. One of these causes, viz., the skill required, connected with the great cost of the material, and consequent high price to the patient, has driven out of general use artificial dentures made of gold or platinum, with porcelain, or of porcelain alone; but the fact still remains that they are the best, in every respect, that can be worn. They have to that extent been transplanted by the vegetable bases which have become so popular within the last twenty-five or thirty years, on account of cheapness and ease of manipulation.

The principal of these are India-rubber (caout-chouc) and cellulose, compounded with other substances to give the former hardness and color, and the latter plasticity under heat, and color. The compounds of these so formed and most used, are hard rubber, or vulcanite and celluloid. The former alone will be spoken of, as it has, since the expiration of the Cummings patent, been rapidly taking the place of the latter, on account of its greater ease of manipulation and its greater durability, and is now so generally used that it may be safely said that there are comparatively few artificial dentures made with any other material. It is admitted by all that the introduction and adoption of this material for dental bases has not been an advance step by the profession, either as regards art or science. It has certainly lowered very much the artistic standard of work in prosthetic dentistry. In a scientific point of view, it has been, in the opinion of a great many of the most prominent members of our profession, productive of serious injury by its effect on the mucous membrane of the mouth and subjacent tissues; so serious that its use has been condemned and its abandonment urged. These opinions, based upon careful observation of facts, have been frequently expressed, since 1869, and can be found on record in the proceedings of dental societies and associations all over the country.

The list of those expressing these opinions is long, and but few will be named. These are Drs. Taft, Atkinson, Chase, Watts, Walker, Cutler. Allen, Eames, Cushing, Morrison, Crouse; all so well known that their names have become familiar as household words in the profession.

The character and symptoms of the pathological conditions (known commonly as rubber sore mouth) produced by the use of rubber plates, have been from time to time described in the proceedings of Dental Societies and Associations, and vary from slight irritation of mucous membrane to ulceration of soft tissues and necrosed bone, with discharge of blood, ichor and pus. One of the worst characteristics of this disease is, that in its progress from simple irritation to ulceration, sloughing and necrosis, it does not, as a general rule, give sufficient pain to warn the patient of danger. The prevalence of these injurious effects may be judged from what Dr. Kulp said before the American Dental Association: "Since the discussion in the Society, I have myself examined 500 cases, and, with the help of some of my friends of the profession, there were in all 1100 cases examined, in three-fourths of which this disease was found;" and from what Dr. W. H. Dorrance said, in a paper read before the same body of the results in 211 partial and full upper cases which he had examined, 165 of which were rubber, and 46 celluloid. In 91 per cent. of these the effects were marked, 47 per cent. of which showed a diseased condition in an aggravated form, and in the remaining nine per cent. there were no effects at all.

Dr. J. R. Walker said before the Southern Dental Association, "That, having satisfied himself of its injurious character, he had abandoned the use of rubber, and called in all his rubber plates for exchange. That this having brought under his observation not only his own patients, but many others; for many years not a day passed that in his office practice he did not see well-defined cases of the specific rubber disease in one form or another, it manifestations being as varied as the old-fashioned liver complaint. Since all the efforts and warnings on the part of so many active and prominent dentists have proved ineffectual to stop or check, to any great extent, the use of a material which produces so generally such baleful effects, the question naturally arises, and it is one which it is the duty of the profession to study and answer. Is there any way to render rubber dental plates harmless, since their use cannot be prevented? Dr. Walker, of New Orleans, Dr. Sturgis, of Quincy, Illinois, and others, found that in cases where the patients, from pecuniary or other reasons, were not willing to discard perfect fitting rubber plates, they cured the disease for the time being, by lining or covering the palatine surface wtth tin or gold foil.

This, however, was only temporary, as the foil as they applied it soon came off. These gentlemen, and all who have tried it, have found that in cases where the disease existed, and had gone no further than to affect the mucous membrane and immediately subjacent tissues, the interposition of a foil of pure metal, which the secretions of the mouth would not change chemically, between the plate and the mucous membrane, cured the disease,

The difficulty with them was, that they could not attach the foil to the plate permanently. This difficulty has been apparently met, as there are now advertised two or three linings for rubber plates, for which it is claimed that they adhere permanently to the rubber.

Here we have the two indispensable conditions complied with; the first in pure gold foil, which undergoes no chemical change in the mouth; the second in the claim that a permanent union can be made between this pure gold and the rubber plate. I have examined one of these advertised linings, and found that the pure gold could not be separated from the rubber by any means, or removed except by scraping, and then a portion of the rubber was scraped off with it.

With this means offered of curing so great an evil, it would seem to be the duty of every dentist to line every new rubber plate that he makes with pure gold, and to examine all those worn by his patients, and apply the lining in every case where the symptoms of the disease appear.—*Dental Office and Laboratory.*

